# BAG2-Mediated Inhibition of CHIP Expression and Overexpression of MDM2 Contribute to the Initiation of Endometriosis by Modulating Estrogen Receptor Status

**DOI:** 10.3389/fcell.2020.554190

**Published:** 2021-04-27

**Authors:** Li-Juan Chen, Bin Hu, Zhi-Qiang Han, Jian-Hua Zhu, Xu Fan, Xue-Xing Chen, Zi-Ping Li, Hao Zhou

**Affiliations:** ^1^Department of Obstetrics and Gynecology, Union Hospital, Tongji Medical College, Huazhong University of Science and Technology, Wuhan, China; ^2^Department of Obstetrics and Gynecology, The Central Hospital of Wuhan, Tongji Medical College, Huazhong University of Science and Technology, Wuhan, China; ^3^Department of Obstetrics and Gynecology, Tongji Hospital, Tongji Medical College, Huazhong University of Science and Technology, Wuhan, China; ^4^Laboratory of Clinical Immunology, Wuhan No. 1 Hospital, Tongji Medical College, Huazhong University of Science and Technology, Wuhan, China; ^5^Beijing Key Laboratory of Translational Medicine in Liver Cirrhosis and National Clinical Research Center of Digestive Diseases, Liver Research Center, Beijing Friendship Hospital, Capital Medical University, Beijing, China; ^6^Union Hospital, Tongji Medical College, Institute of Hematology, Huazhong University of Science and Technology, Wuhan, China

**Keywords:** endometriosis, estrogen receptor, BAG2, CHIP, MDM2, ubiquitin-proteasome pathway

## Abstract

Endometriosis is an estrogen-dependent gynecological disease primarily affecting women of childbearing age, which gives rise to pelvic pain calling for multiple operations, and sometimes leading to infertility. However, the etiology of endometriosis remains poorly understood. In this study we investigated the roles of two Ubiquitin E3 Ligases, namely hsc70-interacting protein (CHIP) and mouse double minute 2 (MDM2), in the abnormal estrogenic activity in endometriosis. We first collected endometrial tissues from 91 cases of endometriosis and 78 cases of uterine myomas. Next, we established a murine endometriosis model by ectopic endometrial tissue implantation. In other studies, we isolated human endometrial stromal cells (HESCs) were isolated from the endometrial tissues, and performed HA- or FLAG-immunoprecipitation assays and immunoblotting with an anti-ubiquitin antibody to test the interactions among BAG2, CHIP, MDM2, estrogen receptor α (ERα), and ERβ. The expression of ERα was downregulated while that of ERβ, BAG2, and MDM2 was upregulated in human endometriosis and in the mouse model. CHIP degraded ERβ instead of ERα *via* the ubiquitin-proteasome pathway, while BAG2 impaired the CHIP-mediated degradation of ERβ in cultured HESCs derived from human endometriosis. The degradation of ERα by MDM2 in cultured endometriosis-HESCs also occurred through the ubiquitin-proteasome pathway. Knockdown of both BAG2 and MDM2 alleviated the development of endometriosis in mice. Our findings suggest that the interference of BAG2 and MDM2 may have therapeutic effects in endometriosis. Understanding better the molecular mechanisms underlying the regulation of the abnormal estrogenic activity in endometriosis is crucial for the advancement of targeted therapeutic strategies.

## Introduction

Endometriosis is a gynecological disease characterized by the abnormal growth of endometrium-like tissue outside the uterine cavity, notably in the ovaries, peritoneum, and rectovaginal septum (Giudice and Kao, 2004; Bulun, 2009). About 7–10% of women of reproductive age suffer from endometriosis, among whom over 50% suffer from pelvic pain (Parazzini et al., 2017). Furthermore, almost half of women with endometriosis may experience infertility caused by impairment of tubal anatomy or function (Bulun et al., 2019). Endometriosis can be diagnosed clinically using gynecologic bimanual examination, recto sigmoidoscopy, barium enema, MRI, or urinary apparatus imaging, followed by confirmation through histological examination (Vercellini et al., 2014). Resections and pharmacological treatment with GnRH agonists are effective in alleviating the pain caused by endometriosis, although often accompanied by hypoestrogenic adverse effects (Brown et al., 2010). Intrauterine insemination and *in vitro* fertilization (IVF) are effective in overcoming infertility caused by endometriosis (de Ziegler et al., 2010). Although various environmental and lifestyle factors are associated with the development of endometriosis, alterations at molecular and cellular levels such as steroid biosynthesis and receptor responses, inflammatory responses as well as increased tissue vascularization, are generally believed to substantially affect the course of endometriosis (Vercellini et al., 2014).

The estrogen class of steroid hormones includes estrone, estradiol (E2), and estriol. Estradiol is the most potent estrogen hormone in circulation, which functions in a wide variety of essential physiological process. Estrogens such as estradiol are critical regulators in the physiological endometrial development under the control of various genes during the menstrual cycle (Kao et al., 2002). Estradiol usually binds to a specific estrogen receptor (ER) that is located in the cytoplasm and in the nucleus of endometrial and other estrogen-responsive cells. The human endometrium expresses two ER subtypes, namely ERα and ERβ, whereby ERα is the primary mediator of physiological responses of Hewitt et al. (2005). Furthermore, previous research shows that ERβ expression exceeds that of ERα in endometrial tissues (Brandenberger et al., 1999). The differential expression of ERα and ERβ in eutopic endometrium and ectopic endometriosis tissue suggests that the two receptors may mediate different functions.

Ubiquitination is a highly conserved process in living organisms that maintains the balance of protein dynamics by programmed degradation. Faulty ubiquitination has also been associated with various disorders, including cancer, metabolic syndromes, and inflammatory conditions (Popovic et al., 2014). Ubiquitination plays an important role in the development of endometriosis through USP10-mediated regulation on the Raf-1/mitogen-activated protein/extracellular signal-regulated kinase (MEK/ERK) pathway (Chen et al., 2018). Bcl2-associated athanogene 2 (BAG2) is a multifunctional co-chaperone that participates in the progression of cancers and some other degenerative diseases (Qin et al., 2016). Although there is no published evidence about the relationship between endometriosis and BAG2, it is known that BAG2 can regulate ubiquitination of target proteins mediated by hsc70-interacting protein (CHIP) (Schonbuhler et al., 2016). Additionally, the mouse double minute 2 (MDM2) protein encoded by the MDM2 gene can promote degradation of the oncogenic p53 protein *via* ubiquitination (Yue et al., 2015). Although the expression of MDM2 is altered in endometriosis, the regulation of disease progression by MDM2 through ER regulation is still not well characterized. Therefore, we investigated in the present study the homeostatic regulation of ER by ubiquitination, focusing on a network involving BAG2 and MDM2, and their effects on the expression of ERβ and ERα.

## Materials and Methods

### Ethics Statement

Study protocols were approved by the Ethics Committee of Tongji Medical College, Huazhong University of Science and Technology and performed in strict accordance with the *Declaration of Helsinki*. All participants signed informed consent documentation prior to sample donation. Animal experiments were approved by the Animal Ethics Committee of Tongji Medical College, Huazhong University of Science and Technology, and were conducted in strict accordance with the Guide for the Care and Use of Laboratory Animals published by the US National Institutes of Health. Extensive efforts were made to ensure minimal suffering of the included animals.

### Tissue Sample Collection

From March 2017 to March 2019, 91 tissue specimens from patients with endometriosis (within the age group of 22–47 years; EMs-H group) and 78 normal endometrial tissue specimens from patients with uterine myoma (Con-H group) were collected the Department of Obstetrics and Gynecology of Central Hospital of Wuhan and Tongji Hospital, Tongji Medical College, Huazhong University of Science and Technology. All patients also donated blood samples. Patients without hormone therapy in the past 6 months prior to the operation, as well as patients with complications such as hypertension, diabetes, and thyroid dysfunction, were excluded from the study. According to the stage of the menstrual cycle and based on hematoxylin-eosin (HE)-staining of tissue sections, 49 patients were in the hyperplastic stage and 42 patients were in the progestational stage. Among tissues in the Con-H group, 43 were in the hyperplastic stage and 35 were in the progestational stage. All the tissue specimens were fixed with 10% formaldehyde, paraffin-embedded, cut into 4 μm sections, where were processed for immunohistochemistry and HE-staining (Sang et al., 2019).

### Ectopic Endometrial Tissue Implantation

Fresh intimal tissue samples were cut into 1–2 mm-thick sections under aseptic conditions. The fragments were cultured in Dulbecco’s modified Eagle’s medium/Ham’s F-12 medium (DMEM/F12) supplemented with 10% fetal bovine serum (FBS) and E2 (10–9 M) for 4 h. Then, ten human endometrial fragments were implanted into the abdominal cavity of female mice with severe combined immunodeficiency disease (SCID) for the establishment of xenograft model of human endometrial carcinoma. The SCID mice were obtained from Beijing Vital River Laboratory Animal Technology Co., Ltd. (Beijing, China). Four weeks after implantation, the mice were euthanized by intraperitoneal (i.p.) injection of 3% pentobarbital sodium (P3761, Sigma-Aldrich Chemical Company, St. Louis, MO, United States), and tissues removed to observe the pathological changes of endometriosis. To identify the overexpression or loss of BAG2/MDM2 in xenograft mice, Con-M mice were assigned randomly to groups consisting of overexpression-negative control (oe-NC) and HA-MDM2 + FLAG-BAG2 groups, and En-M mice were assigned to short hairpin RNA (sh)-NC and sh-BAG2 + sh-MDM2 groups. At 24 h after xenotransplantation, mice received i.p. intraperitoneal of 10 μL adenoviruse (1 × 10^10^ PFU/mL) after an interval of 1 day. Four weeks after this injection, seven mice in each group were euthanized for subsequent experiments.

### Cell Culture

Human endometrial stromal cells (HESCs) were isolated from endometrial tissues derived from patients with endometriosis (HESCs-En) and patients with uterine myomas (HESCs-NC). The tissues were mechanically dispersed with a scalpel, detached with 0.1% type I collagenase and 0.05% DNAase at 37°C for 1 h, and then resuspended in the growth medium. The mixture of endometrial cells (epithelial cells and stromal cells) was passed through a 40 μm sieve to separate the smaller stromal and larger epithelial cells (Millipore, Billerica, MA, United States). The stromal cells passing through the sieve were collected and resuspended in growth medium consisting of DMEM/F12 (1:1), 10% FBS, 1% penicillin/streptomycin, and 1% amphotoxicin B. HEK293 cells purchased from American Type Culture Collection (Manassas, VA, United States) were added to the Petri dish, cultured in the cell culture box and sub-cultured three times. The detailed method for stromal cell culture is as described previously (Hu et al., 2012).

### Cell Treatment

Lentiviruses and adenoviruses were purchased from Sangon Biotech (Shanghai, China). The primer sequences and plasmid construction were also performed by Sangon Biotech, and cell transfection was carried out according to their instructions. In the transfection for 48 h transfection, Lipofectamine 2000 reagents (Invitrogen Inc., Carlsbad, CA, United States) were used as per the manufacturer’s instructions.

### Determination of Estradiol Concentration

Culture medium was collected from the blood samples or treated cells after 48 h of transfection, followed by steroid extraction using phenol. The concentrations of estradiol in the extracts were determined with an enzyme-linked immunosorbent assay (ELISA) kit (Cayman Chemical, Ann Arbor, MI, United States) following the manufacturer’s protocol.

### Reverse Transcription Quantitative Polymerase Chain Reaction (RT-qPCR)

The total RNA from tissues or cells was extracted with TRIzol reagents (16096020, Thermo Fisher Scientific Inc., Waltham, MA, United States) and reverse transcribed into complementary DNA (cDNA) using the reverse transcription kit (4368813, Thermo Fisher Scientific Inc., Waltham, MA, United States). The RT-qPCR kit (11732020, Thermo Fisher Scientific Inc., Waltham, MA, United States) was employed to perform RT-qPCR assay following the manufacturer’s protocol. The mRNA expression of each sample was normalized to glyceraldehyde-3-phosphate dehydrogenase (GAPDH) mRNA. Relative mRNA expression was measured using the formula 2^–ΔΔC^. The primer sequences were synthesized by TaKaRa company (Takara Holdings Inc., Kyoto, Japan) (Table 1). Each reaction was performed in triplicate.

**TABLE 1:** Primer sequences of related genes for RT-qPCR.

Gene	Primer sequence
ERα-H	F: 5′-CCACCAACCAGTGCACCATT-3′
ERα-H	R: 5′-GGTCTTTTCGTATCCCACCTTTC-3′
ERβ-H	F: 5′-AGAGTCCCTGGTGTGAAGCAAG-3′
ERβ-H	R: 5′-GACAGCGCAGAAGTGAGCATC-3′
ERα-M	F: 5′-CGTGTGCAATGACTATGCCTCT-3′
ERα-M	R: 5′-TGGTGCATTGGTTTGTAGCTGG-3′
ERβ-M	F: 5′-GTCAGGCACATCAGTAACAAGGG-3′
ERβ-M	R: 5′-ATTCAGCATCTCCAGCAGCAGGTC-3′
MDM2-M	F: 5′-GTCTGTGTCTACCGAGGGTG-3′
MDM2-M	R: 5′-TAAGTGTCGTTTTGCGCTCC-3′
MDM2-H	F: 5′-GGCTTTGATGTTCCTGAT-3′
MDM2-H	R: 5′-TTGTCTTGGGTTTCTTCC-3′
BAG2-H	F: 5′-CTTTGAGAGAAGCAGCAACTG-3′
BAG2-H	R: 5′-TGACACTTCAACGGTGAGAG-3′
BAG2-M	F: 5′-AGACGCAGCTACTGCTGTTG-3′
BAG2-M	R: 5′-CGGATCGTTTCCACCGAGAC-3′
GAPDH-H	F: 5′-TCCCATCACCATCTTCC-3′
GAPDH-H	R: 5′-GAGGCTGTTGTCATACTTCT-3′
GAPDH-M	F: 5′-GCAAAGTGGAGATTGTTGCCAT-3′
GAPDH-M	R: 5′-CCTTGACTGTGCCGTTGAATTT-3′

*F, forward; R, reverse; H, human; M, mouse.*

### Western Blot Analysis

The total protein of the cells was extracted according to the manufacturer’s instructions of the protein extraction kit (BC3640, Beijing Solarbio Science and Technology Co., Ltd., Beijing, China). The protein content was then quantified using a bicinchoninic acid (BCA) kit (20201ES76, Yeasen Biotechnology Co., Ltd., Shanghai, China). The total protein was separated by 10% sodium dodecyl sulfate-polyacrylamide gel electrophoresis, followed by electro-transfer onto a nitrocellulose membrane. The membrane was incubated with monoclonal antibodies to β-tubulin (ab6046, 1:5,000), MDM2 (ab38618, 1:1,000), BAG2 (PA5-78853, 1:1,000), ERβ (ab3576, 1:1,000), ERα (ab32063, 1:1,000), HA (ab9110, 1:1,000), Myc (ab32, 1:1,000), FLAG (ab125243, 1:1,000), and Ubiquitin (ab7780, 1:1,000) overnight at 4°C. The following day, the membrane was further incubated with secondary antibody to rabbit/mouse antibody to immunoglobulin G (IgG) conjugated with horseradish peroxidase (1:2,000, ab6721/ab6728) for 1 h at 37°C. All the aforementioned antibodies were purchased from Abcam Inc. (Cambridge, MA, United States), except for BAG2, which was purchased from Invitrogen Inc. (Carlsbad, CA, United States). The immune complexes were detected using enhanced chemiluminescence (ECL) detection reagents (Pierce, Waltham, MA, United States) at room temperature for 1 min. Finally, the solution was discarded and the membrane was covered with plastic wrap and exposed to an Image Quant LAS 4000C gel imager (General Electric Company, Boston, MA, United States). The relative protein was quantified on band volume with respect to β-tubulin expression.

### Immunofluorescence Staining

The cells were grown on confocal culture dishes and transfected with plasmids. After 24 h of transfection, the cells were fixed with 4% paraformaldehyde for 10 min and permeabilized with Triton-X buffer containing 50 mM Tris-HCl (pH = 7.5), 0.5% Triton X-100, 150 mM NaCl and 2 mM EDTA for 15 min. The cells in each well were blocked with PBS containing 1% bovine serum albumin (BSA) and 0.5% goat serum for 3 h at 37°C. The cells were incubated with the primary antibody in PBS containing 1% BSA for 2 h at 37°C. Afterward, the cells were incubated with Alexa-Fluor-594-conjugated rabbit/mouse antibody to IgG (1:500, ab150077/150120; Abcam Inc., Cambridge, MA, United States) for 1 h at 37°C. Finally, the cells were mounted with 4′, 6-diamidino-2-phenylindole (DAPI), dried, and photographed under a fluorescence microscope (IX73, Olympus Optical Co., Ltd., Tokyo, Japan).

### Co-immunoprecipitation (Co-IP) Assay

At 48 h after transfection, the cells were placed on ice for another 48 h and then lysed with IP lysis buffer, triturated evenly with a pipette, and transferred to Eppendorf (EP) tubes. After being lysed on ice for 30 min, the cells were centrifuged at 14,800 rpm for 20 min at 4°C. The supernatant was then transferred to a new EP tube and the protein concentration was measured by the BCA method. An 1 mg portion of protein was taken for each sample, added with IP lysis buffe, and made up to 500 μL. Samples in each tube were then incubated at 4°C overnight with primary antibodies. On the next day, the sample in each tube was further incubated with 20 μL Protein A + G beads for 2 h, then eluted 5 times with IP lysis buffer with centrifugation at 2,500 rpm for 5 min at 4°C. After the removal of the supernatant, cells were denatured for 5 min at 100°C with 20 μL loading buffer (2×).

### Ubiquitin Detection

The amount of samples binding to IP was analyzed by immunoblotting with antibody to Ubiquitin (ab7780, 1:1,000, Abcam Inc., Cambridge, MA, United States).

### Immunohistochemical Staining

The paraffin sections of the mouse endometrial tissues were heated at 60°C for 20 min to facilitate dewaxing, and then placed in xylene (15 min each time), and transferred to in anhydrous alcohol for 5 min, followed by 95 and 70% alcohol (10 min each time). The endogenous peroxidase was inactivated by incubation in 3% H_2_O_2_ for 10 min. An antigen-presenting step was performed by steaming the sections in sodium citrate buffer for 3 min. After pre-incubation with 2% normal goat serum (Shanghai Sangon Biological Engineering Technology and Services Co., Ltd., Shanghai, China) at room temperature for 20 min, the sections were incubated with primary antibodies to MDM2 (ab38618, 1:500), BAG2 (PA5-78853, 1:500), ERβ (ab3576, 1:500), ERα (ab32063, 1:500) overnight at 4°C, followed by incubation with biotin-conjugated goat anti-rabbit antibody to IgG (1:500, ab97049) for 30 min. All antibodies were purchased from Abcam Inc., (Cambridge, MA, United States), except the BAG2 antibody, which was purchased from Invitrogen Inc. (Carlsbad, CA, United States). After incubation with SABC (Vector Laboratories, Inc., Burlingame, CA, United States) in a 37°C incubator for 30 min, the sections were developed with reagent A, B, and C in the diaminobenzidine (DAB) color development kit (Sigma-Aldrich Chemical Company, St Louis, MO, United States) for 6 min. The sections were counterstained with hematoxylin for 30 s, dehydrated, cleared, and mounted for observation and photography under an inverted microscope (IX73, Olympus Optical Co., Ltd., Tokyo, Japan). To analyze semi-quantitatively the immunoreactivity of nuclear steroid receptors we used an H score, with more than 500 ESCs: 0 indicates no staining, 1 indicates moderate, and 2 indicates high staining. The H-score was then calculated by multiplying the number of each point with the percentage of that point (Wu et al., 2017).

### Statistical Analysis

Statistical analysis was carried out using the SPSS 21.0 software (IBM Corp., Armonk, NY, United States). Data were expressed as mean ± standard deviation. Data obeying normal distribution and homogeneity of variance between two groups were compared using unpaired *t*-test. The data with skewed distribution or unequal variances were compared by the rank sum test. A value of *p* < 0.05 was considered statistically significant.

## Results

### Content of Estradiol and Expression Levels of ERβ and ERα in Human and Mouse Endometriosis

**FIGURE 1:**
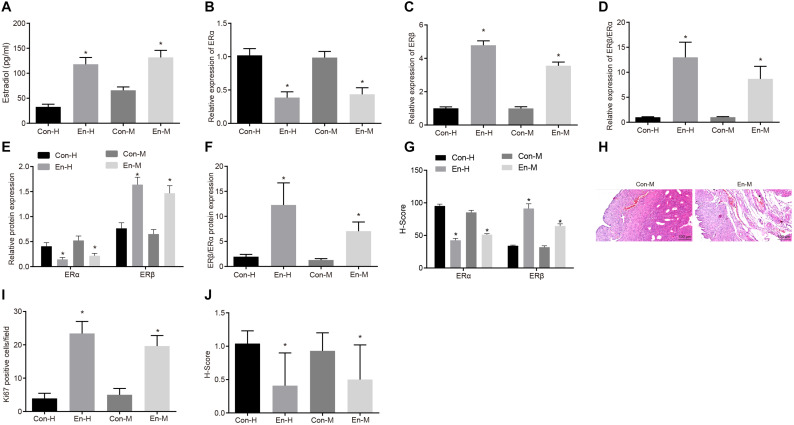
Content of estradiol and expression levels of ERβ and ERα in human and mouse endometriosis. **(A)** The expression of estradiol in human and mouse endometriosis/normal tissues determined by ELISA. **(B)** The mRNA expression of ERα in human and mouse endometriosis/normal tissues determined by RT-qPCR. **(C)** The mRNA expression of ERβ in human and mouse endometriosis/normal tissues determined by RT-qPCR. **(D)** The relative expression of ERβ/ERα in human and mouse endometriosis/normal tissues determined by RT-qPCR. **(E)** The protein expression of ERα and ERβ in human and mouse endometriosis/normal tissues determined by Western blot analysis. **(F)** The protein expression of ERβ/ERα in human and mouse endometriosis/normal tissues determined by Western blot analysis. **(G)**, The statistical analysis of immunohistochemical staining of ERα and ERβ in human and mouse endometriosis/normal tissues. **(H)** HE staining of mouse endometriosis and normal tissues. **(I)**, The statistical analysis of fluorescence staining of Ki67 in human and mouse endometriosis/normal tissues. **(J)** The statistical analysis of immunohistochemical staining of cleaved caspase-8 in human and mouse endometriosis/normal tissues. Comparisons between two groups were analyzed using independent sample *t*-test. *p < 0.05 vs. the human or mouse normal tissues. *n* = 78 for human normal tissues; *n* = 91 for human endometriosis tissues; *n* = 14 for mouse endometriosis/normal tissues.

To investigate the expression of estradiol, ERα and ERβ in clinical samples and mouse models of endometriosis, we performed ELISA, RT-qPCR, Western blot analysis, and immunohistochemical staining. The ELISA results showed that the estradiol expression was higher in human endometriosis tissues than that in normal human intimal tissues, and likewise for the mouse samples (both *p* < 0.05; Figure 1A). RT-qPCR and Western blot analysis revealed down-regulation of ERα mRNA (*p* < 0.05; Figure 1B) and protein (*p* < 0.05; Figure 1E) expression in human and mouse endometriosis tissues, and up-regulation of ERβ mRNA (*p* < 0.05; Figure 1C) and protein (*p* < 0.05; Figure 1E) expression relative to normal human and mouse intimal tissues, thereby giving an increased ratio of ERβ/ERα mRNA (*p* < 0.05; Figure 1D) and protein expression (*p* < 0.05; Figure 1F). The same trend was observed in immunohistochemical staining (Figure 1G and Supplementary Figure 1A). The results of HE staining demonstrated that the mouse model of endometriosis was successfully induced (Figure 1H). The Ki67 fluorescence staining showed that the number of Ki67 positive cells was higher in human and mouse endometriosis tissues than that in normal human and mouse intimal tissues, respectively (*p* < 0.05; Figure 1I and Supplementary Figure 1B). On the other hand, the immunohistochemical staining of cleaved caspase-8 protein showed the opposite changes (*p* < 0.05; Figure 1J and Supplementary Figure 1C). In summary, estradiol and ERβ were highly expressed while ERα had low expression, both in both clinical samples and in tissues from the mouse endometriosis models.

### ERα Is Down-Regulated and ERβ Is Up-Regulated in HESCS-En *in vitro*

**FIGURE 2:**
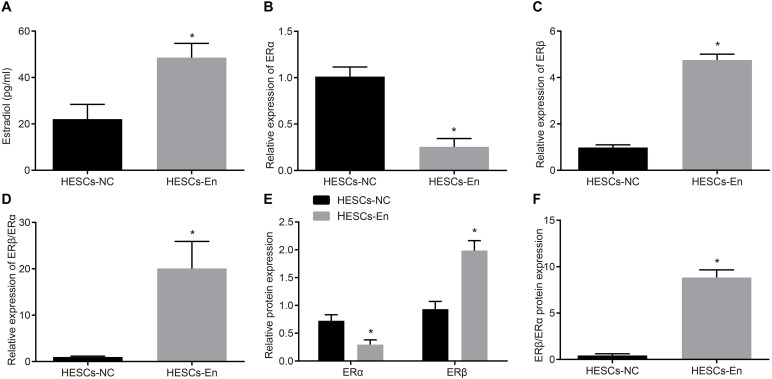
ERα was down-regulated and ERβ was up-regulated in endometriosis-related HESCs *in vitro*. **(A)** The expression of estradiol in HESCs-NC and HESCs-En determined by ELISA. **(B)** The mRNA expression of ERα in HESCs-NC and HESCs-En determined by RT-qPCR. **(C)** The mRNA expression of ERβ in HESCs-NC and HESCs-En determined by RT-qPCR. **(D)** The relative expression of ERβ/ERα in HESCs-NC and HESCs-En determined by RT-qPCR. **(E)** The protein expression of ERα and ERβ in HESCs-NC and HESCs-En determined by Western blot analysis. **(F)** The protein expression of ERβ/ERα in HESCs-NC and HESCs-En determined by Western blot analysis. Comparisons between two groups were analyzed using independent sample *t*-test. *p < 0.05 vs. the HESCs-NC cells. Cell experiments were conducted in triplicate.

The expressions of ERα and ERβ in HESCs-En were measured by RT-qPCR and Western blot analysis, which showed higher. the content of estradiol in the HESCs-En than in the HESCs-NC (Figure 2A). RT-qPCR and Western blot analysis also revealed down-regulation of ERα mRNA (*p* < 0.05; Figure 2B) and protein (*p* < 0.05; Figure 2E) expression in HESCs-En, while showing up-regulation of ERβ mRNA (*p* < 0.05; Figure 2C) and protein (*p* < 0.05; Figure 2E) relative to HESCs-NC. These effects propagated to higher expression ratios for ERβ/ERα mRNA (*p* < 0.05; Figure 2D) and the corresponding protein (*p* < 0.05; Figure 2F), thus indicating successful formation of the endometriosis phenotype.

### CHIP Degrades ERβ Instead of ERα by Ubiquitin-Proteasome Pathways in HESCs *in vitro*

**FIGURE 3:**
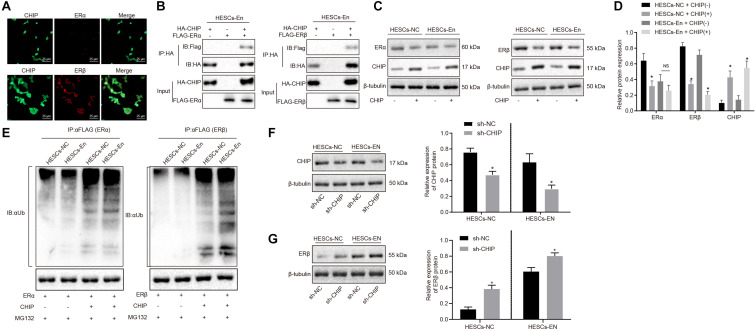
CHIP degraded ERβ by ubiquitin-proteasome pathways in HESCs *in vitro*. **(A)** The distribution of CHIP, ERα and ERβ in the cells. **(B)** The interaction of CHIP, ERα and ERβ in HESCs-En cells by Co-IP. **(C)** The protein expression of ERα and ERβ in HESCs-NC and HESCs-En cells measured by Western blot analysis. **(D)** The statistical analysis of panel C. **(E)** Ubiquitin of ERα and ERβ proteins in HESCs-NC and HESCs-En cells by ubiquitin assay. **(F)**| The protein expression of CHIP following sh-NC and sh-CHIP treatment measured by Western blot analysis. **(G)** The protein expression of ERβ following sh-NC and sh-CHIP treatment measured by Western blot analysis. Comparisons between two groups were analyzed using independent sample *t*-test. *p < 0.05 vs. the sh-NC or HESCs-NC + CHIP^–^; ^#^*p* < 0.05 vs. HESCs-En + CHIP^–^. Cell experiments were conducted in triplicate.

ERα and ERβ ubiquitin are degraded through the ubiquitin proteasome pathway by the E3 ubiquitin ligase, known as CHIP protein. To investigate whether CHIP might be responsible for the reduced expression of ERα and the relative enhancement of ERβ expression, overexpression vectors for ERα or ERβ and CHIP were introduced into HEK293 cells. After 48 h of treatment, the results of immunofluorescence staining showed that ERα and ERβ were mainly located in the nucleus and that CHIP protein was found both in cytoplasm and nucleus (Figure 3A). Then, to verify the interactions between CHIP and ERα/ERβ, HESCs-En cells were treated with HA-CHIP, FLAG-ERβ, HA-CHIP, and FLAG-ERα plasmids, respectively. The interaction between CHIP and ERα, and between CHIP and ERβ were detected by the Co-IP test using HA antibody (Figure 3B).

In HESCs-NC and HESCs-En cells, Western blot analysis showed that the protein expression of ERα and ERβ was down-regulated by CHIP^+^ vs. CHIP^–^ in HESCs-NC cells (*p* < 0.05). However, in HESCs-En cells, the protein expression of ERα showed no significant difference, while that of ERβ was abrogated by CHIP^+^ relative to CHIP^–^ cells (*p* < 0.05; Figures 3C,D). In HESCs-NC cells, the ubiquitination of ERα and ERβ proteins was augmented by CHIP^+^ vs. CHIP^–^ (*p* < 0.05; Figure 3E), while in HESCs-En cells, CHIP^+^ failed to enhance ERα ubiquitin, but restored that of ERβ compared to CHIP^–^ (*p* < 0.05; Figure 3E). For further verification, lentivirus packaging was used to deliver sh-CHIP into HESCs-NC and HESCs-En cells. As shown in Figures 3F,G, the results showed that the protein level of CHIP (Figure 3F) was decreased and the protein level of ERβ (Figure 3G) was increased following sh-CHIP treatment. These results imply that CHIP mediates the degradation of ERα and ERβ in HESCs-NC cells. However, in HESCs-En cells, CHIP rather mediates the degradation of ERβ instead of ERα. Thus, we speculate that the expression of ERα may be regulated by other E3 ubiquitin ligases.

### BAG2 Inhibits CHIP-Mediated Degradation of ERβ in HESCs *in vitro*

**FIGURE 4:**
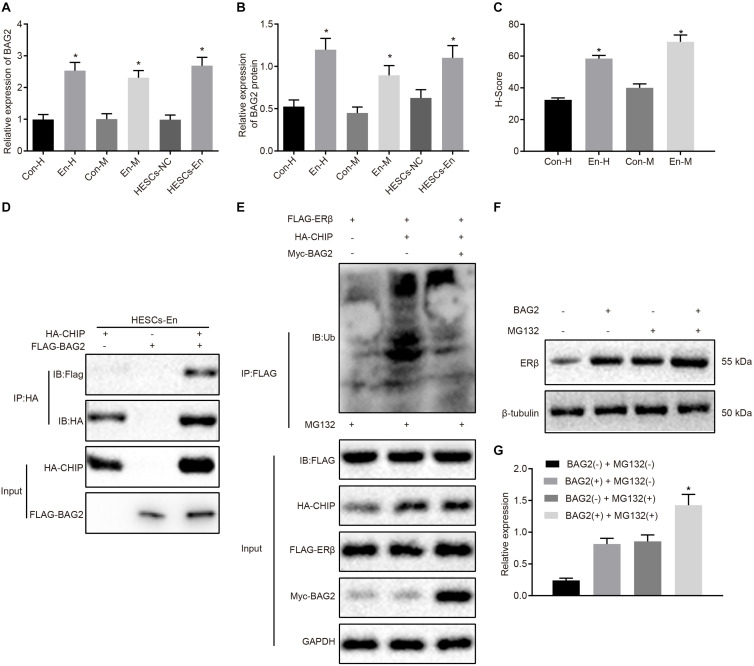
BAG2 inhibits CHIP-mediated degradation of ERβ in HESCs *in vitro*. **(A)** The mRNA expression of BAG2 in human and mouse endometriosis/normal tissues and HESCs determined by RT-qPCR. **(B)** The protein expression of BAG2 in human and mouse endometriosis/normal tissues and HESCs determined by Western blot analysis. **(C)** The statistical analysis of immunohistochemical staining of BAG2 in human and mouse endometriosis/normal tissues. **(D)** The interaction between CHIP and BAG2 by Co-IP assay. **(E)** Ubiquitin of ERβ in the presence or absence of BAG2 by ubiquitin test. **(F)** The protein expression of ERβ in the presence or absence of BAG2 by Western blot analysis. **(G)** The statistical analysis of panel **(F)**. Comparisons between two groups were analyzed using independent sample *t*-test. *p < 0.05 vs. human or mouse normal tissues, HESCs-NC cells or the absence of BAG2. *n* = 78 for human normal tissues; *n* = 91 for human endometriosis tissues; *n* = 14 for mouse endometriosis/normal tissues. Cell experiments were conducted in triplicate.

The above results showed that ERβ was degraded by CHIP *via* ubiquitin, while the expression of ERβ was up-regulated in endometriosis. Therefore, we postulated that there may be other factors inhibiting the degradation of ERβ by CHIP. BAG2 has been documented previously to act as a molecular chaperone of the CHIP protein and to inhibit the E3 ubiquitin ligase function of CHIP protein (Arndt et al., 2005). Therefore, we speculated that BAG2 may increase the expression of ERβ by inhibiting the ubiquitination by CHIP of ERβ. To test this hypothesis, we measured the expression of BAG2 in human and mouse endometriosis/normal tissues and in the HESCs. The results of RT-qPCR (Figure 4A) and Western blot analysis (Figure 4B) showed that the mRNA and protein expression of BAG2 in endometrial tissues from human endometriosis (En-H tissues) and from mouse endometriosis (En-M tissues), as well as in HESCs-En cells, were up-regulated when compared with endometrial tissues from human uterine myomas (Con-H) and from healthy normal mice (Con-M) tissues, as well as in HESCs-NC cells (*p* < 0.05). Matching results were obtained using immunohistochemistry (*p* < 0.05; Figure 4C and Supplementary Figure 2A). After overexpressing CHIP and BAG2 in HEK293 cells for 48 h, we conducted immunofluorescence staining, which showed similar cellular distributions of BAG2 and CHIP proteins (Supplementary Figure 2B). Co-IP revealed that the interaction between BAG2 and CHIP was detected with an HA antibody (Figure 4D). To investigate further that overexpression of ERβ resulted from the inhibitory effect of BAG2 on the ubiquitination of CHIP, we used anti-FLAG antibody in the Co-IP assay and then applied ubiquitin antibody in immunoblotting. The results showed that the ubiquitination of ERβ was reduced (Figure 4E), while the protein expression was enhanced (Figures 4F,G) in the presence of BAG2, thus suggesting that BAG2 can suppress the CHIP-dependent degradation of ERβ.

### Degradation of ERα Is a Consequence of MDM2 Ubiquitin in HESCs *in vitro*

**FIGURE 5:**
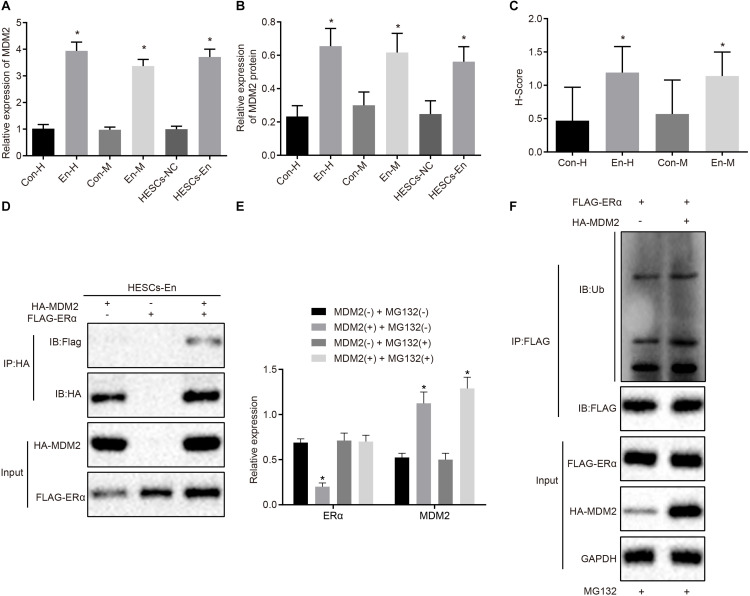
MDM2 decreases the transcriptional activity of ERα by degradation in HESCs *in vitro*. **(A)** The mRNA expression of MDM2 in human and mouse endometriosis/normal tissues and HESCs determined by RT-qPCR. **(B)** The protein expression of MDM2 in human and mouse endometriosis/normal tissues and HESCs determined by Western blot analysis. **(C)** The statistical analysis of immunohistochemical staining of MDM2 in human and mouse endometriosis/normal tissues. **(D)** The interaction between MDM2 and ERα by Co-IP assay. **(E)** The protein expression of ERα and MDM2 in the presence or absence of MDM2 by Western blot analysis. **(F)** Ubiquitin of ERα in the presence or absence of MDM2 by ubiquitin test. Comparisons between two groups were analyzed using independent sample *t*-test. *p < 0.05 vs. human or mouse normal tissues, HESCs-NC cells or MG132-treated cells without MDM2. *n* = 78 for human normal tissues. *n* = 91 for human endometriosis tissues. *n* = 14 for mouse endometriosis/normal tissues. Cell experiments were conducted in triplicate.

A previous study has shown an association between high MDM2expression with the occurrence of endometriosis (Sang et al., 2019). Besides, E3 ubiquitin ligase MDM2 could mediate the degradation of ERα *via* the ubiquitin-proteasome pathway (Tateishi et al., 2004; Duong et al., 2007). Therefore, the mRNA and protein expression of MDM2 was determined in the human and mouse endometriosis/normal tissues and in the HESCs. The RT-qPCR results (Figure 5A) and Western blot analysis (Figure 5B) showed that the mRNA and protein expression of MDM2 in En-H and En-M tissues, as well as HESCs-En cells, was up-regulated compared with Con-H and Con-M tissues, as well as HESCs-NC cells (*p* < 0.05). The same results were obtained using immunohistochemistry (*p* < 0.05; Figure 5C and Supplementary Figure 2C). We thus speculated that MDM2 in HESCs-NC and HESCs-En cells may be ubiquitinated to degrade ERα, and tested the localization of MDM2 and ERα in HEK293 cells by transferring HA-MDM2 and FLAG-ERα overexpression plasmids. After 48 h of treatment, immunofluorescence staining showed that MDM2 and ERα were located in the nucleus (Supplementary Figure 2D). In an attempt to confirm the interaction between MDM2 and ERα, we overexpressed MDM2 and ERα overexpressed in HESCs-En cells, and detected the binding of MDM2 and ERα by Co-IP assay (Figure 5D).

To detect whether MDM2-mediated ubiquitin can lead to the degradation of ERα, we performed Western blot analysis in HESCs-En cells. The results demonstrated that the expression of ERα was reduced by MDM2^+^ compared to MDM2− group (*p* < 0.05; Figure 5E), which was related to the ubiquitin protease pathway. We used the FLAG antibody in the Co-IP assay and ubiquitin antibody in the immunoblotting reaction, finding that ubiquitination of ERα was enhanced in the presence of MDM2 (Figure 5F). Overall, these results suggest that decreased ERα protein expression in HESCs-En cells may be related to MDM2.

### BAG2-Mediated Elevation of ERβ and MDM2-Mediated Inhibition of ERα Contribute to Endometriosis Development

**FIGURE 6:**
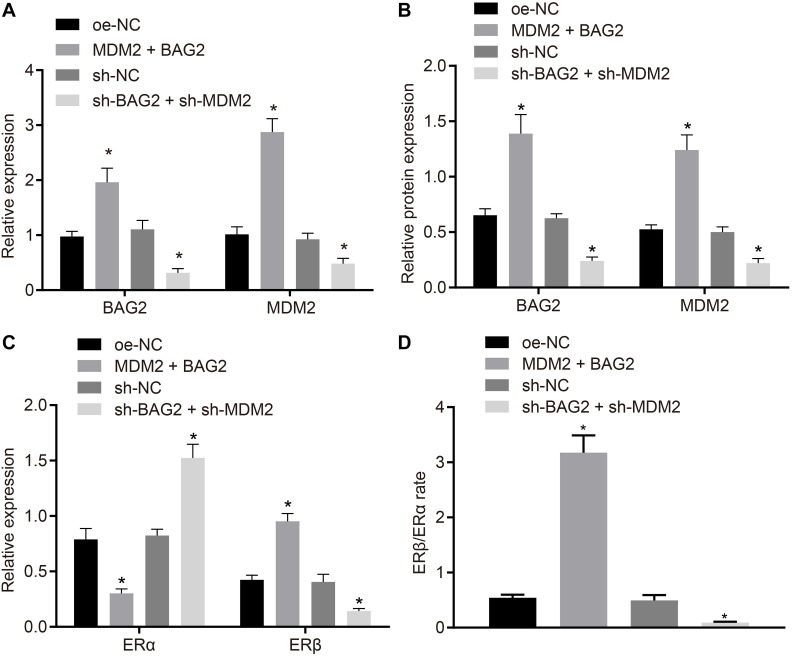
BAG2-mediated inhibition of ERα and MDM2-mediated elevation of ERβ contributes to the development of endometriosis. HESCs-NC cells were treated with overexpressed MDM2 and BAG2 with oe-NC as control, while HESCs-En cells were treated with sh-BAG2 and sh-MDM2 with sh-NC as control. **(A)** The mRNA expression of BAG2 and MDM2 determined by RT-qPCR. **(B)** The protein expression of BAG2 and MDM2 determined by western blot analysis. **(C)** The protein expression of ERα and ERβ determined by western blot analysis. **(D)** The protein expression of ERβ/ERα determined by western blot analysis. Comparisons between two groups were analyzed using independent sample *t*-test; *p < 0.05 vs. oe-NC treatment; ^#^*p* < 0.05 vs. sh-NC treatment. Cell experiments were conducted in triplicate.

To verify the effect of BAG2 and MDM2 on the expression of ERα and ERβ, MDM2 and BAG2 were overexpressed in HESCs-NC cells, while the expression of MDM2 and BAG2 were silenced in the HESCs-En cells. After 48 of treatment, the mRNA expression of BAG2 and MDM2 was detected by RT-qPCR, while the protein expression of BAG2, MDM2, ERα and ERβ was detected by Western blot analysis in cells. RT-qPCR demonstrated that the mRNA expression of BAG2 and MDM2 was up-regulated in HESCs-NC with MDM2 and BAG2 overexpression when compared to cells treated with oe-NC (*p* < 0.05; Figure 6A). In contrast, the mRNA expression of BAG2 and MDM2 was reduced in the shBAG2 + shMDM2-treated HESCs-En cells when compared to treatment with sh-NC (*p* < 0.05; Figure 6A). The results of Western blot analysis showed that the protein expression of ERα (Figure 6C) was lower in HESCs-NC cells overexpressing MDM2 and BAG2 than that in HESCs-NC cells overexpressing oe-NC, while that of BAG2, MDM2 (Figure 6B) and ERβ (Figure 6C), as well as the ratio of ERβ/ERα (Figure 6D), were increased (*p* < 0.05). These results indicated the successful formation of the endometriosis phenotype. As expected, silencing of both BAG2 and MDM2 in HESCs-En cells led to opposite effects (*p* < 0.05).

### Knockdown of BAG2 and MDM2 Reduces the Occurrence of Endometriosis in Mice

**FIGURE 7:**
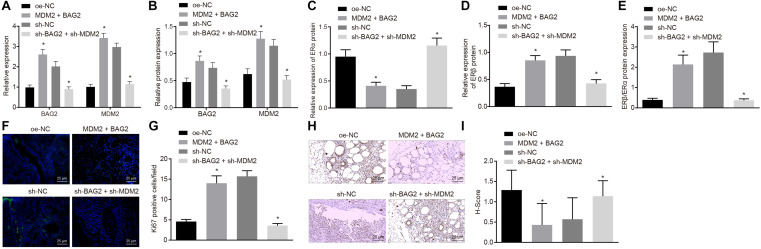
The interference of BAG2 and MDM2 showed therapeutic effects on mouse endometriosis. Con-M mice were treated with overexpressed MDM2 and BAG2 using oe-NC as control, while En-M mice were treated using sh-BAG2 and sh-MDM2 with sh-NC as control. **(A)** The mRNA expression of BAG2 and MDM2 determined by RT-qPCR. **(B)** The protein expression of BAG2 and MDM2 determined by Western blot analysis. **(C)** The protein expression of ERα determined by Western blot analysis. **(D)** The protein expression of ERβ determined by Western blot analysis. **(E)** The protein expression of ERβ/ERα determined by Western blot analysis. **(F)** Fluorescence staining of Ki67 in mouse tissues. G, The statistical analysis of **(F)**. **(H)** Immunohistochemical staining of cleaved caspase 8 in mouse tissues. **(I)** The statistical analysis of panel **(H)**. Comparisons between two groups were analyzed using independent sample *t*-test. *n* = 8. *p < 0.05 vs. oe-NC treatment; ^#^*p* < 0.05 vs. sh-NC treatment.

To evaluate the effects of BAG2 and MDM2 *in vivo*, we established a mouse model of endometriosis and detected. The expression of BAG2, MDM2, ERα, and ERβ in mouse endometrium was detected by RT-qPCR and Western blot analysis. The results of RT-qPCR showed that the mRNA expression of BAG2 and MDM2 was up-regulated in Con-M mouse tissues following MDM2 + BAG2 treatment compared to oe-NC treatment (*p* < 0.05; Figure 7A). In En-M mouse tissues, the mRNA expression of BAG2 and MDM2 was reduced by sh-BAG2 + sh-MDM2 treatment when compared to the sh-NC treatment (*p* < 0.05; Figure 7A). The results of Western blot analysis revealed that the protein expression of ERα (Figure 7C) was lower in tissues of Con-M mice overexpressing MDM2 and BAG2 than that in tissues of Con-M mice overexpressing oe-NC, while that of BAG2, MDM2 (Figure 7B) and ERβ (Figure 7D), as well as the ratio of ERβ/ERα (Figure 7E) was increased (*p* < 0.05). Again, these results were consistent with successful formation of endometriosis phenotype. In addition, simultaneous silencing of BAG2 and MDM2 had the opposite effects in En-M mice (*p* < 0.05). Results obtained from Ki67 fluorescence staining illustrated that, in Con-M mouse tissues, the number of Ki67 positive cells was increased following MDM2 and BAG2 overexpression when compared to oe-NC treatment (*p* < 0.05; Figures 7F,G). Furthermore, in En-M mouse tissues, the number of Ki67 positive cells was much lower in the absence of both BAG2 and MDM2, than upon silencing of NC (*p* < 0.05; Figures 7F,G). Consistent with these results, concomitant overexpression of MDM2 and BAG2 down-regulated cleaved caspase-8 expression in Con-M mouse tissues as revealed by immunohistochemical staining, whereas concomitant silencing of MDM2 and BAG2 up-regulated cleaved caspase-8 expression in En-M mouse tissues (*p* < 0.05; Figures 7H,I).

## Discussion

Irrespective of the existing therapies including drugs, surgery and combined treatment, the clinical recurrence rate of endometriosis is high, and there is an absence of satisfactory treatment for this recurrence (Sang et al., 2019). To improve the efficiency of endometriosis treatments, it is of great importance to uncover the unique molecular properties of endometriotic tissues relative to normal endometria (Han et al., 2015). In the present study, we have demonstrated that the expression of ERα was lower, while that of ERβ was higher in clinical samples of endometriosis and in the mouse model. Besides, we found that BAG2 and MDM2 were both elevated in the endometriosis tissues compared with normal endometrium. We further determined that BAG2 could inhibit CHIP ubiquitin ligase activity, thus alleviating ERβ degradation, while high expression of MDM2 promoted ubiquitin-dependent degradation of ERα.

Recently, the regulatory role of estrogen on tumor microenvironment has drawn the attention of a great number of researchers, and ERβ has been reported to be upregulated in the tumor-associated macrophages of ovarian cancer (Jing et al., 2019). The growth suppressor action of ERβ and the reduced expression of ERβ were found in association with carcinogenesis, indicating a tumor suppressor role in epithelial ovarian cancer and malignant gliomas (Treeck et al., 2019). Conversely, endometriotic lesions resulted in alteration of the ERα and ERβ expression ratios when compared to that in eutopic endometrium, while both ERs were present in uterine and various immune cell types involving neutrophils and macrophages (Burns et al., 2018). The mRNA expression of ERα has been reported to be remarkably enhanced in endometriotic lesions and eutopic endometrium compared to that of ERβ (Bukulmez et al., 2008). However, when co-expressed, ERβ exerts an inhibitory effect on ERα-modulated transcription, such that the efficiency of estrogens and/or their receptors is dependent on the balance of ERα and ERβ, instead of on their respective expression rates (Linares et al., 2017). Additionally, in the ventral lobes of the prostate of rats, the mRNA expression ratio of ERβ/ERα was markedly reduced when compared to that normal rats (Mizoguchi et al., 2017). In contrast, the present study revealed that the expression of ERα was diminished, while the expression of ERβ was promoted in endometriosis. In line with our findings, previous research shows that the conjugated estrogen/bazedoxifene tissue-selective estrogen complex induced ERα degradation through the F-box protein 45/ubiquitin proteasome, resulting in attenuated ERα activity both in endometrial and breast cells (Han et al., 2016).

Moreover, the ubiquitination pathway is extensively involved in the regulation of ERα and ERβ expression in endometriosis. Previously, Zhao et al. (2015) revealed that Diptoindonesin G could mediate ER protein stability, thus hindering the transcriptional activity of ERα and restoring the transcriptional activity of ERβ by interacting with the CHIP E3 ubiquitin ligase. ERα and ERβ share three common ubiquitin E3 ligases, i.e., MDM2, E6AP, and CHIP E3 ligase, such that targeting CHIP E3 ubiquitin ligase facilitates the regulation of Diptoindonesin G in ERβ stability (Zhao et al., 2015). Notably, silencing of Pescadillo resulted in elevated ubiquitination of endogenous ERα, whereas reduced ubiquitination of endogenous ERβ in MCF7 cells by the CHIP-modulated ubiquitin-proteasome pathway was observed (Cheng et al., 2012). BAG-2 was identified as a main component of CHIP complexes, and BAG-2 can inhibit the ubiquitin ligase activity of CHIP by abrogating the CHIP/E2 cooperation (Arndt et al., 2005), which is in line with our present findings. The age-induced enhancement of BAG2 expression effectively abrogates the activity of the E3 ubiquitin ligase, which contributes to the downregulation of CHIP-regulated ubiquitination of heat shock protein 72 (Schonbuhler et al., 2016). Thus, the functional relevance of the BAG2/CHIP/ERβ axis is substantiated. Furthermore, long-term ovariectomy results in increased ubiquitination of ERα and degradation due by the targeted proteasome, while ERα expression could be restored by suppressing the proteasome (Zhang et al., 2011). Besides, increased expression of MDM2 has been corroborated in endometriosis and is reportedly is closely linked to the dysfunction of normal endometrium, cell cycle, as well as the progression of endometriosis (Sang et al., 2019). Moreover, the positive expression rate of MDM2 protein was much higher in ovarian endometriosis than that in normal endometrium, thus participating in the pathogenesis and development of endometriosis (Sang et al., 2019). MDM2 induces a significant enhancement in ERα-mediating gene expression and estrogen responsiveness through interactions with ERα in MCF-7 and ZR-75 breast cancer cells (Kim et al., 2011). A previous study also reported that overexpression of MDM2 leads to enhanced ERα ubiquitination (Duong et al., 2007). Also, the E3 ligases MDM2 and CHIP are reported to be associated with ERα signaling, and MDM2-related ubiquitination plays a vital role in ERα regulation of Cav1.2 in ovariectomized APP/PS1 mice (Lai et al., 2019).

**FIGURE 8:**
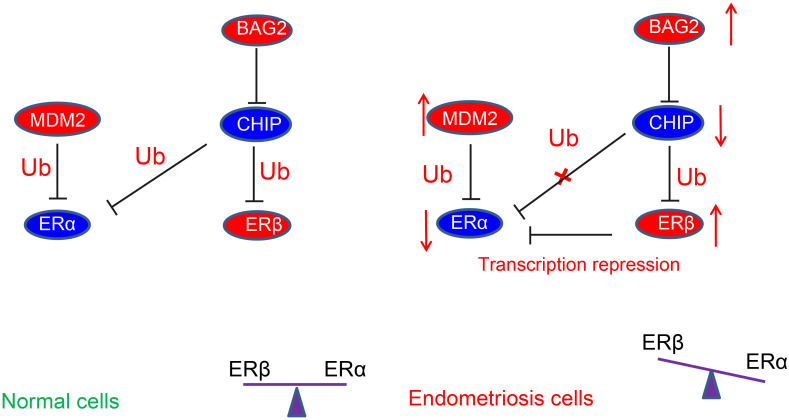
Schematic diagram depicting the regulatory mechanism underlying abnormal estrogenic activity in endometriosis. The high expression of BAG2 in the endometrium reduces the degradation of ubiquitinated ERβ by CHIP, thus increasing the protein expression of ERβ. On the other hand, the high expression of MDM2 increases the degradation of ubiquitinated ERα and diminishes the protein expression of ERα, leading to a notable increase of ERβ/ERα ratio, thereby inducing endometriosis.

In conclusion, the current study demonstrated that overexpression of BAG2 in the endometrium reduced the ubiquitination of ERβ by CHIP and potentiate the protein expression of ERβ. On the other hand, overexpression of MDM2 enhanced the degradation of ubiquitination of ERα and diminished the protein expression of ERα, thus contributing to the development of endometriosis (summarized in Figure 8). These results suggest new potential treatment targets for endometriosis. A more detailed investigation on the specific ER agonists or androgen receptor antagonists could elucidate the receptor-specific mechanisms and their interactions in the modulation of endometriosis.

## Data Availability Statement

The original contributions presented in the study are included in the article/Supplementary Material, further inquiries can be directed to the corresponding author/s.

## Ethics Statement

Written informed consent was obtained from all patients prior to the study. Study protocols were approved by the Ethics Committee of Tongji Medical College, Huazhong University of Science and Technology and followed the tenets of the Declaration of Helsinki. Animal experiments were conducted in strict accordance with the Guide to the Management and Use of Laboratory Animals issued by the National Institutes of Health. All the animals’ experiments were done at the animal center of Tongji Medical College, Huazhong University of Science and Technology, and the protocol of animal experiments was approved by the Animal Ethics Committee of Tongji Medical College, Huazhong University of Science and Technology.

## Author Contributions

L-JC, BH, Z-QH, and J-HZ conceived and designed the research. Z-QH, J-HZ, and XF performed the experiments. X-XC, Z-PL, and HZ analyzed the data. L-JC and BH interpreted the results of experiments. J-HZ and XF prepared the figures. BH, X-XC, and Z-PL drafted the manuscript. L-JC, Z-QH, and HZ edited and revised the manuscript. L-JC, BH, Z-QH, J-HZ, XF, X-XC, Z-PL, and HZ approved final version of the manuscript. All authors contributed to the article and approved the submitted version.

## Conflict of Interest

The authors declare that the research was conducted in the absence of any commercial or financial relationships that could be construed as a potential conflict of interest.

## Supplementary Material

Supplementary Figure 1 Representative images of immunohistochemical staining and fluorescence staining in Figure 1. **(A)** The immunohistochemical staining of ERα and ERβ in human and mouse endometriosis/normal tissues. **(B)** The fluorescence staining of Ki67 in human and mouse endometriosis/normal tissues. **(C)** The immunohistochemical staining of cleaved caspase-8 in human and mouse endometriosis/normal tissues.

Supplementary Figure 2 Representative images of immunohistochemical staining and fluorescence staining in Figures 4, 5. **(A)** The immunohistochemical staining of BAG2 in human and mouse endometriosis/normal tissues. **(B)** The fluorescence staining of BAG2 and CHIP in cells. **(C)** The immunohistochemical staining of MDM2 in human and mouse endometriosis/normal tissues. **(D)** The fluorescence staining of MDM2 and ERα in cells.
