# Supplementary figures and images for: BAG2-Mediated Inhibition of CHIP Expression and Overexpression of MDM2 Contribute to the Initiation of Endometriosis by Modulating Estrogen Receptor Status
Source: Front Cell Dev Biol. 2021 Apr 27;8:554190. doi: 10.3389/fcell.2020.554190 (PMC8111302; doi:10.3389/fcell.2020.554190)

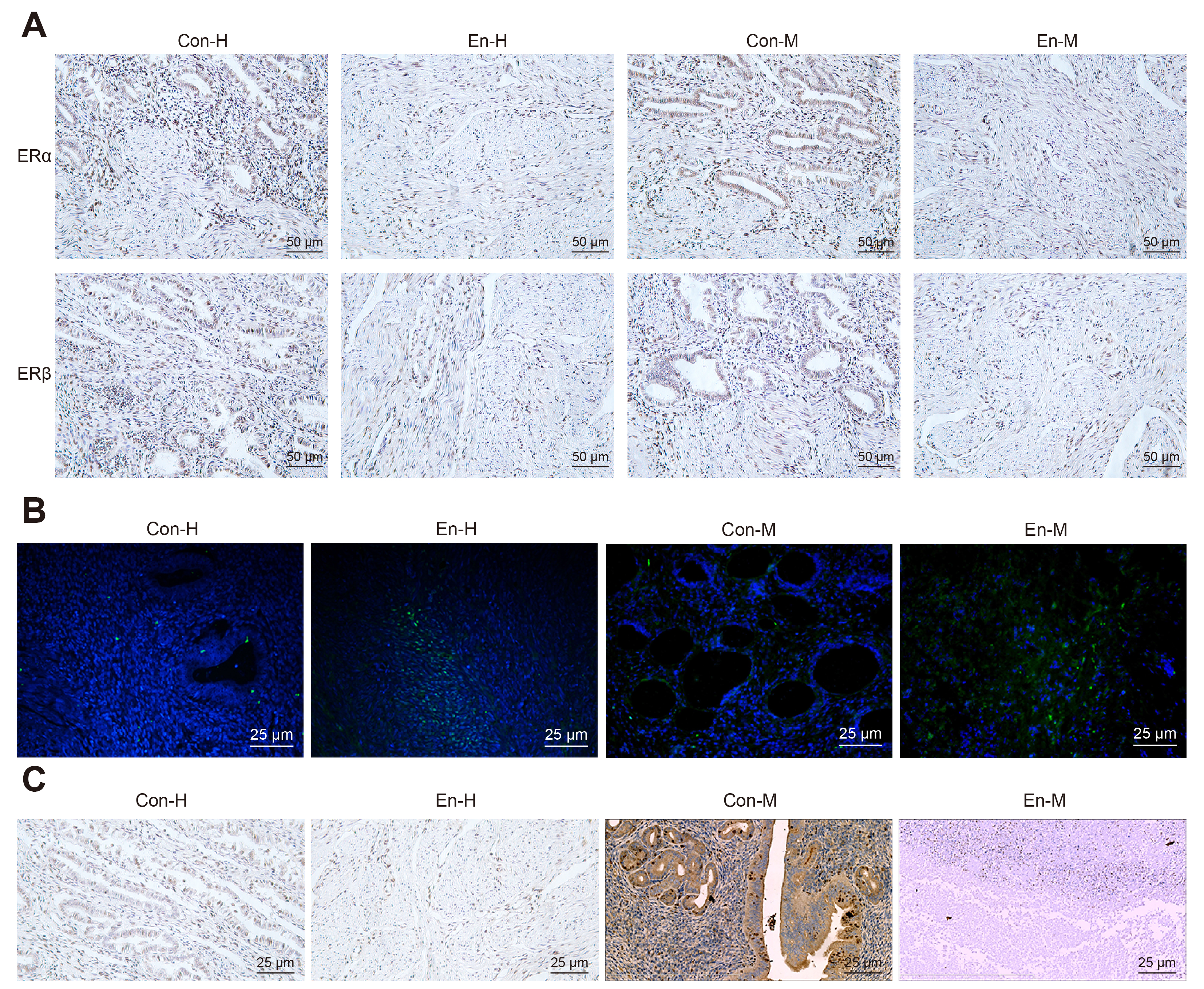

Supplement: Supplementary Figure 1 — Representative images of immunohistochemical staining and fluorescence staining in Figure 1. (A) The immunohistochemical staining of ERα and ERβ in human and mouse endometriosis/normal tissues. (B) The fluorescence staining of Ki67 in human and mouse endometriosis/normal tissues. (C) The immunohistochemical staining of cleaved caspase-8 in human and mouse endometriosis/normal tissues. [file Image_1.jpg]

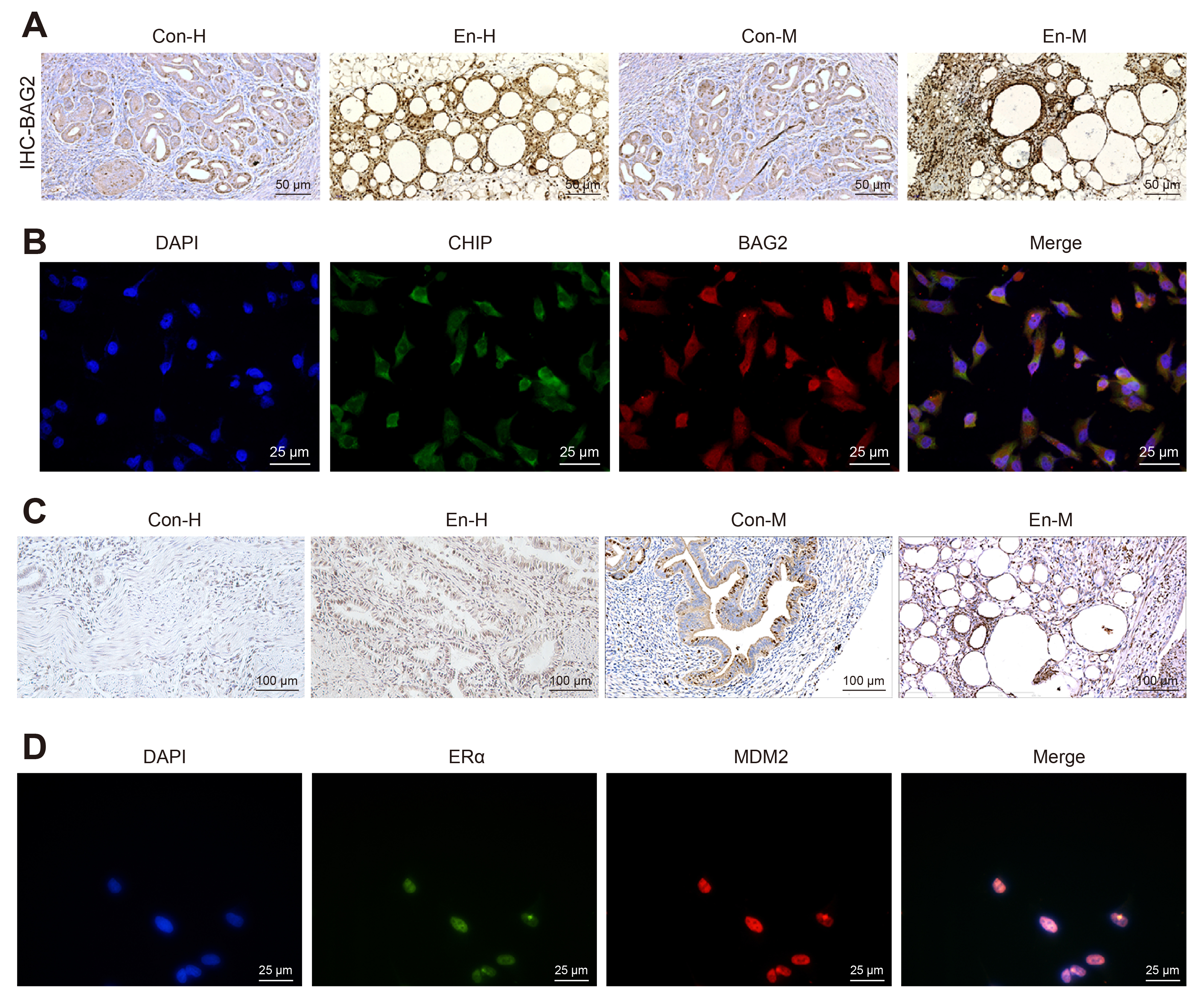

Supplement: Supplementary Figure 2 — Representative images of immunohistochemical staining and fluorescence staining in Figures 4, 5. (A) The immunohistochemical staining of BAG2 in human and mouse endometriosis/normal tissues. (B) The fluorescence staining of BAG2 and CHIP in cells. (C) The immunohistochemical staining of MDM2 in human and mouse endometriosis/normal tissues. (D) The fluorescence staining of MDM2 and ERα in cells. [file Image_2.jpg]
